# Oncological Impact of Surgical Margin Status After Transoral Laser Microsurgery for Early Glottic Cancer

**DOI:** 10.1002/hed.70013

**Published:** 2025-08-11

**Authors:** Sae Hatomi, Shuta Tomisato, Ken Kasahara, Hiroyuki Ozawa, Takeyuki Kono

**Affiliations:** ^1^ Department of Otolaryngology‐Head and Neck Surgery Keio University School of Medicine Tokyo Japan

**Keywords:** glottic cancer, prognosis, recurrence, surgical margin, transoral laser microsurgery

## Abstract

**Background:**

Transoral laser microsurgery is a well‐established treatment for early glottic cancer. However, managing patients with close/positive margins remains debated. This study analyzes the relationship between resection margins and oncologic outcomes.

**Methods:**

Patients with early glottic cancer who underwent transoral laser microsurgery were included. We compared clinical characteristics, recurrence‐free survival, laryngeal preservation, and disease‐specific survival between negative and close/positive margin groups.

**Results:**

Among 107 patients, 49 (45.8%) had close/positive margins. The recurrence‐free survival rate was significantly lower in the close/positive margin group. Anterior commissure involvement was a significant risk factor for recurrence. The median recurrence time for the close/positive margin group was 18.5 months, similar to the negative margin group. Laryngeal preservation and disease‐specific survival were similar between groups with > 95%.

**Conclusions:**

Patients with close/positive margins in early glottic cancer showed similar disease‐specific survival and laryngeal preservation rates as negative margins; despite lower recurrence‐free survival, suggesting a follow‐up approach is sufficient.

## 1 Introduction

Transoral laser microsurgery (TLM) is a well‐established minimally invasive treatment approach for early glottic squamous cell carcinoma (SCC). According to the National Comprehensive Cancer Network (NCCN) guidelines, Tis, T1, T2, and selected T3 glottic cancers are good indications for TLM, in which short hospitalization and low complication rates have been noted [1, 2, 3]. At the same time, inadequate laryngeal exposure, paraglottic space involvement with arytenoid fixation, massive pre‐epiglottic space invasion, and laryngeal framework erosion may be considered reasonable limitations [4]. When performing TLM, oncological and functional outcomes after treatment need to be accounted for, considering that the balance between these two aspects is critical to achieving optimal goals. We perform phonosurgical narrow cancer‐free margin resection using intraoperative narrow‐band image (NBI) evaluation, which can penetrate superficial vascular structures and facilitate the detection of malignant lesions [5], and preserve as much normal tissue as possible. Previous studies have proven that TLM via NBI promoted a substantially lower positive margin and recurrence rates than did TLM via white light observation [6, 7]. Our previous comparative multidimensional analysis on laryngeal function after radiotherapy (RT) and TLM for early glottic cancer revealed that the phonosurgical resection technique successfully promotes equivalent post‐therapeutic voice when compared to RT based on subjective and objective evaluations [8]. However, this conservative approach has the potential to increase the incidence of close or positive margins following resection. Thus, the following three options have been proposed for managing cases with suspected positive margins: (1) additional treatment including TLM reintervention or RT; (2) a second endoscopic evaluation 6–8 weeks after TLM to determine the need for reintervention; (3) a follow‐up approach involving placing particular attention to suspicion of recurrence/progression based on videolaryngostroboscopy. Managing patients with inadequate surgical margins is still a matter of ongoing discussion, with no common consensus regarding further treatment having yet been established.

Elucidating the oncological progression in cases with positive or close margins is clinically crucial for determining the necessity of additional treatment. In instances of close or positive margins following TLM, we employ a follow‐up strategy, intervening only when clear signs of recurrence are observed. The present study retrospectively examines the association between resection margins and long‐term oncologic outcomes in patients with early glottic cancer who underwent TLM.

## 2 Methods

### 2.1 Study Design and Patients

We included a total of 107 patients with Tis‐selected T2 glottic cancer who underwent TLM using a CO_2_ laser at our institution between January 2012 and September 2023. Patients who could not be followed for more than 1 year after surgery were excluded. Moreover, those whose cancer was obviously not resected based on intraoperative findings and additional treatment performed shortly after TLM were also excluded. Ethical approval for this study was obtained from the Ethics Committee of the Keio University School of Medicine (approval number: 20200127). The committee waived the need for informed consent for the publication of anonymized information in this article.

### 2.2 Surgical Procedure, Pathological Evaluations, and Follow‐Up Schedule

**FIGURE 1:**
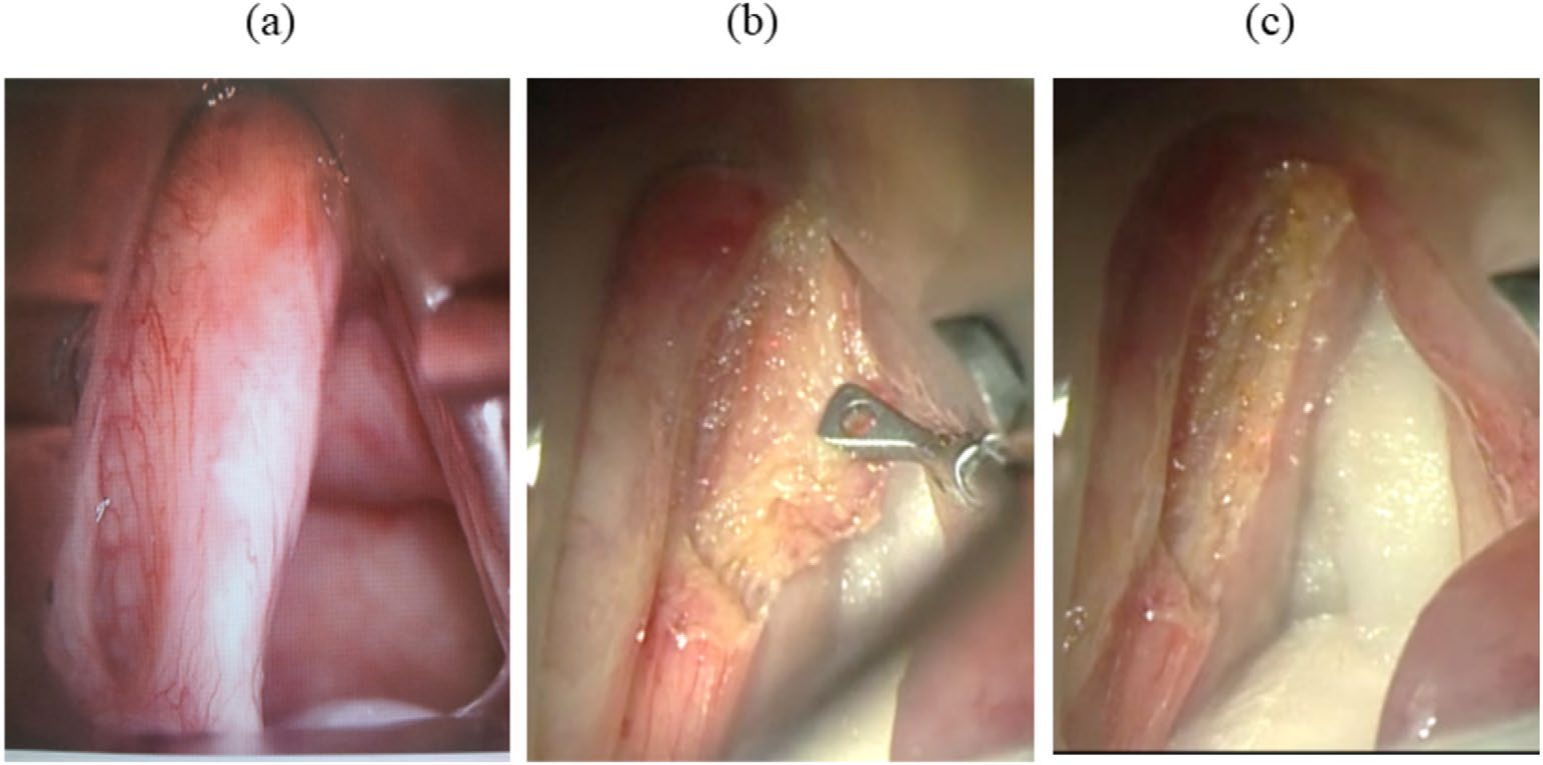
Intraoperative findings of phonological laser cordectomy. Pre‐ (a), intra‐ (b), and post‐operative (c) findings are shown. The microflap technique allows complete tumor resection without carbonizing the deep layer. [Color figure can be viewed at wileyonlinelibrary.com]

Endoscopic CO_2_ laser surgery was performed using the Lumenis Acupulse with AcuBlade (Lumenis Ltd., Yokneam, Israel). We used the focused dot beam spot with a diameter of approximately 240 μm to achieve a cutting effect. The laser was used in the superpulse continuous mode, and the AcuBlade joystick was used to direct the scan pattern onto the tissue target, striving to irradiate the area within a short period of time with rapid scanner movements to reduce thermal damage to peripheral tissues. This procedure enables precise stripping of the tumor using a phonosurgical microflap technique without carbonizing the adjacent tissue (Figure 1). The tumor was resected using the en bloc technique based on type II, III, and IV cordectomies according to the European Laryngological Society (ELS) classification [9]. Gross resection of the tumor was completed through intraoperative observation using the NBI mode of the flexible endoscope. In patients with T1b glottic cancer who exhibited tumors on both sides of the vocal cords, the surgery was performed in two phases to prevent laryngeal web formation, with each surgery being considered a separate case.

Pathohistological and immunohistochemical analyses of the surgical specimens were performed by pathologists. Positive margins were defined as resection margins that are in close proximity to tumor (< 1 mm) or are positive for cancer or atypical cells.

Follow‐up schedules were based on NCCN guidelines. Briefly, patients underwent videolaryngostroboscopy coupled with NBI every month during the first year, every 2 months during the second year, and every 3–6 months until the fifth year after TLM [10]. Those who wanted to were followed up for approximately 10 years after surgery.

### 2.3 Data Collection and Statistical Analysis

Clinical data, such as age, gender, observation period, smoking history, T staging, tumor localization, types of cordectomy, presence of prior treatment, and recurrence status, were retrospectively collected from the medical records.

Statistical analysis was performed using IBM SPSS Statistics version 29.0 (IBM, Armonk, NY), with the results being expressed as mean ± SD. Statistically significant differences were determined using the paired Student's t‐test. Recurrence‐free survival (RFS) among patients with negative and close/positive margins was estimated using the Kaplan–Meier method, with intergroup differences being determined using the log‐rank test. Moreover, binomial logistic regression was performed to identify risk factors affecting local recurrence among patients with close/positive margins. Fisher's exact test was applied to compare the laryngeal preservation rate (LPR) and disease‐specific survival (DSS) among the groups. In all analyses, a *p*‐value of < 0.05 indicated statistical significance.

## 3 Results

**FIGURE 2:**
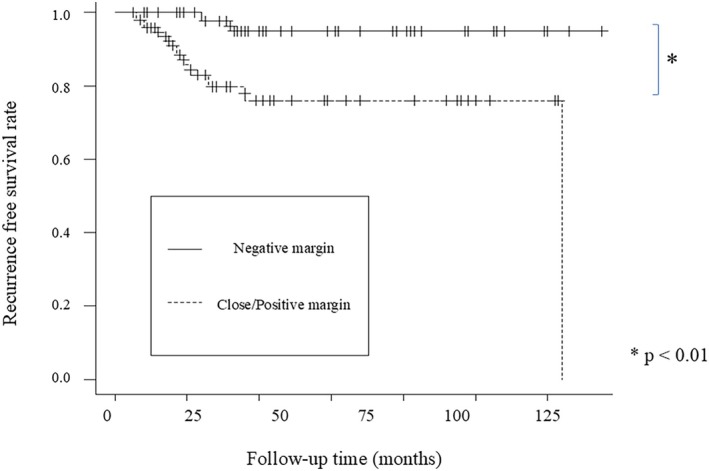
RFS rates of negative and close/positive margin group. RFS rate was significantly lower in close/positive margin group (bold line) than negative margin group (thin line). [Color figure can be viewed at wileyonlinelibrary.com]

This retrospective study included 97 men and 10 women with a mean age of 71.0 ± 9.2 years (range, 38–87 years). The follow‐up duration was 15–135 months (54.0 ± 35.7 months). Type II, III, and IV cordectomy was performed in 86, 15, and 6 cases, respectively, according to the ELS classification of laser cordectomy. A total of 20 patients underwent TLM as salvage surgery. Of these, 13 patients initially received (chemo)radiotherapy as the primary treatment, while 7 patients were treated exclusively with transoral surgery. Moreover, surgical margins were negative in 58 patients and close or positive in the remaining 49 patients, indicating that 45.8% of the patients had unsafe margins. Details regarding the characteristics of each group are presented in Table 1. No significant differences in clinical backgrounds were observed between the close/positive and negative margin groups. Local recurrence was observed in two cases with negative margins (2/58, 3.4%) and in 10 cases with close/positive margins (10/49, 20.4%). Kaplan–Meier analysis revealed that the RFS rate was significantly lower in the close/positive margin group than in the negative margin group (*p* < 0.01; Figure 2).

**TABLE 1:** Patient characteristics of positive margins group and negative margins group.

	All patients (*n* = 107)	Positive margins (*n* = 49)	Negative margins (*n* = 58)	*p*‐value
Gender				
Male	97	47	50	0.08
Female	10	2	8	
Age (years)	71 ± 9.2 (38–87)	71 ± 9.4 (50–87)	70 ± 9.1 (38–85)	0.701
Follow‐up (months)	54 ± 35.7 (15–135)	51 ± 35.5 (15–124)	56 ± 35.9 (15–135)	0.417
Initial treatment				
Yes	87	36	51	0.056
No	20	13	7	
Smoking				
Yes	88	41	47	0.72
No	19	8	11	2
Staging				
Tis	17	9	8	0.68
T1a	67	28	39	7
T1b	12	7	5	
T2	11	5	6	
Cordectomy				
Type II	86	40	46	0.931
Type III	15	6	9	
Type IV	6	3	3	

**FIGURE 3:**
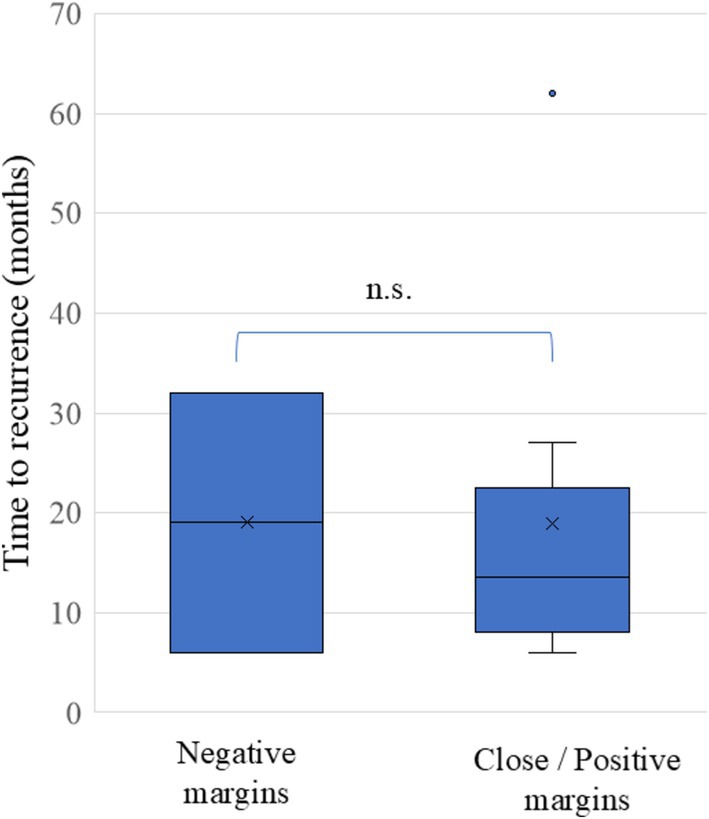
The duration to recurrence after TLM. There were no significant differences in the duration of recurrence between close/positive (median 18.5 months) and negative margin groups (median 18.5 months). [Color figure can be viewed at wileyonlinelibrary.com]

To identify additional independent risk factors for local recurrence following TLM, a binomial logistic regression analysis was conducted. The independent variables included gender, age, the Brinkman index (calculated by multiplying the number of cigarettes smoked per day by the number of years an individual has smoked) to assess smoking history, a history of prior RT, and involvement of the anterior commissure (AC). Notably, only AC involvement was identified as a significant risk factor for recurrence in the close/positive margin group (odds ratio, 27.882; 95% CI, 3.023–257.141; *p* = 0.003, Table 2). The median time to recurrence was 18.5 months (range, 9–63 months) and 19 months for the close/positive and negative margin groups, respectively, with no significant differences between the two groups (Figure 3). Among 12 patients with local recurrence after TLM, two refused further treatment due to advanced age and succumbed to the disease. Three patients with a history of RT underwent further total laryngectomy, and two patients with T1b cancer underwent further RT. The remaining 5 patients underwent additional TLM, all of whom were successfully managed following second‐line treatment.

**TABLE 2:** Risk factors for recurrences after transoral laser microsurgery.

Factors	Odds ratio (95% CI)	*p*‐value
Gender	0	0.999
Age	1.006 (0.922–1.098)	0.896
Brinkman index	1.000 (0.998–1.002)	0.822
Radiotherapy	2.596 (0.323–20.899)	0.370
AC involvement	27.882 (3.023–257.141)	0.003

The LPR was 98.3% (57/58) and 95.9% (47/49) in the negative and close/positive margin groups, respectively, with no significant difference between the groups (*p* = 0.330). We performed a subgroup analysis stratifying patients according to whether TLM was used as first‐line or second‐line treatment. In the first‐line treatment group, the LPR was 100% irrespective of surgical margin status. In the second‐line treatment group, the LPR was 84.6% (11/13) in positive‐margin cases and 85.7% (6/7) in negative‐margin cases. These findings were consistent with the overall results of the study and further support the conclusion that surgical margin status has no significant impact on laryngeal preservation. The DSS rates for the negative and close/positive margin groups were 98.3% (57/58) and 98.0% (48/49), respectively, with no significant difference between the groups (*p* = 1.0).

## 4 Discussion

Previous studies revealed that TLM demonstrated comparable or superior oncologic outcomes compared to RT among patients with early glottic cancer, including higher DSS rates, local control rates, and LPR. Large cohort studies have shown local control rates exceeding 90%, with even better 5‐year DSS and LPR at 95% [11, 12, 13, 14, 15]. Despite favorable survival outcomes, TLM has exhibited recurrence rates of 10%–31% [11, 16, 17, 18, 19]. Several studies examining the association between margin status and recurrence rates after TLM have reported a positive margin rate of 9.3%–45.4% as well as a recurrence rate of 8%–51% and 3.1%–22.8% among those with a positive and negative margin, respectively [20, 21, 22, 23, 24]. Our study found a positive margin rate of 45.8% and a recurrence rate of 20.4% and 3.4% in the close/positive and negative margin groups, respectively. Compared to published literature, our study showed a slightly higher positive margin rate but a similar local recurrence rate. Several explanations can clarify why not all patients with positive margins develop recurrences. Margin assessment is difficult and often inaccurate due to the combination of ultra‐narrow surgical margins (typically 1–3 mm), specimen orientation issues, laser coagulation artifacts at the margins, and specimen shrinkage due to the presence of contractile proteins in the connective tissue and their release from surrounding structures. The literature reports a mean mucosal specimen shrinkage of 3.8 ± 0.3 mm in the anteroposterior length of the glottic plane after TLM, from intralaryngeal measurement to post‐resection [25]. This shrinkage is evidenced by the high rates of unevaluable or indeterminate margins after TLM, ranging from 17.2% to 33% [26], and is believed to be partially responsible for the high rates of apparently unsafe margins, with studies reporting close and positive margin rates of 50% [27]. Moreover, up to 80% of positive and close margins on definitive pathology can be considered false positives as evidenced by the high rates of negative second‐look TLM procedures [2, 28].

Numerous studies have shown that positive margins were an independent risk factor for local recurrence [21, 27, 29]. Long‐term analyses on glottic SCC support the utility of re‐resection following TLM in cases with positive margins given the relatively low recurrence rate after re‐resection compared to after close observation approaches [30]. Meanwhile, margin status did not appear to negatively affect 5‐year overall survival (OS), DSS, and LPR [12, 31]. According to the large sample study by the National Cancer Database, patients with positive margins and those with negative margins after TLM for early glottic SCC demonstrated similar 5‐year OS [26]. Based on available data, the ELS guidelines have supported the follow‐up and re‐evaluation of patients with laryngeal cancer who have positive margins [32]. Our study revealed that the close or positive margin group had a significantly lower RFS than did the negative margin group (*p* < 0.01), but no significant difference in DSS (*p* = 1.0) and LPR (*p* = 0.33) were observed between the groups.

AC involvement has also often been suggested to be a risk factor for local recurrence. In the management of tumors involving the AC, complete exposure, appropriate endoscopic and radiologic evaluation, and advanced surgical skills are essential. Regarding surgical skills, the learning curve for TLM is an important issue owing to the limited surgical field view [33]. Although some previous studies have reported that AC involvement did not contribute to recurrence [15, 34], several recent studies have reported that AC inclusion significantly increases the risk of recurrence depending on T staging [31, 35, 36]. Notably, AC involvement was associated with decreased local control in T1a and T1b but not in T2 tumors, although analysis of 463 T1–T2 patients showed that AC involvement had no impact on survival [35]. In contrast, Hakeem found that local control was significantly lower in T2 patients with AC involvement but not in those with T1a or T1b tumors, although LPR and OS remained unaffected in 296 T1–T2 patients [37]. In a study involving 201 patients with Tis–T2 glottic cancer, patients with AC involvement had poor local control and lower LPR and DSS rates [36].

Rucci proposed a specific classification of AC involvement, namely AC1, AC2, and AC3, wherein AC1 involves only one side of the midline, AC2 involves a subsite crossing the midline on only part of the longitudinal extension, and AC3 involves the entire AC subsite bilaterally across the midline [38]. Carta et al. reported that although AC1 and AC2 did not significantly affect patient prognosis, AC3 patients showed a poorer 5‐year RFS (74.1%) [39]. Horseshoe tumors with AC involvement typically originate in one vocal cord and migrate horizontally to the opposite vocal cord. These tumors have less potential to infiltrate the cartilage via perichondrial dehiscence at Broyles' ligament and have a better prognosis than tumors arising from the AC, which extend predominantly vertically [40]. Our study revealed that although AC involvement significantly increased the risk of local recurrence among the positive margin group, it did not affect survival. Therefore, these data suggest that TLM still remained an effective treatment option for tumors located in the AC given that this minimally invasive transoral approach is repeatable and maintains optimal DSS even after multiple interventions.

Avoiding unnecessary surgical procedures has the advantage of preserving voice quality given that repeat TLM promotes further tissue loss and increases scarring of the remaining vocal cords. Moreover, even minor surgical procedures entail the use of healthcare resources, incur financial costs, and may require hospitalization, representing a potentially nontrivial burden for some patients. Considering that only 20% of our patients with positive margins experienced local recurrence and that margin status did not affect the DSS rate and LPR, our findings suggest that the majority of patients with positive margins may have been overtreated with revision surgery. Furthermore, those with positive margins and AC involvement in our study had a median time to recurrence of approximately 1.5 years, which suggests a particularly high risk of recurrence. Regarding follow‐up protocols, the relatively frequent surveillance schedule employed in this study adhered to the NCCN guidelines, which recommend examinations every 1–3 months in the first year and every 2–6 months in the second year. We believe that this approach contributed to the early detection of recurrences and the high laryngeal preservation rates observed in our cohort. However, this intensity of follow‐up may differ from routine practice in some institutions, potentially limiting the generalizability of our findings. Based on our experience and the observed recurrence patterns, we propose that follow‐up intervals could be tailored according to surgical margin status: patients with negative margins may be safely monitored at longer intervals, while those with positive or close margins may benefit from more frequent follow‐up within the recommended ranges to facilitate early identification of recurrences.

Our study has several limitations. First, the small number of patients in each subgroup may have introduced bias and confounding that may not have been adequately addressed in the analysis. As a result, the statistical power was insufficient to detect significant differences. Second, information on the orientation of the margins could not be obtained in all cases. Third, the location of the margins, such as superficial and deep layers, could further enhance the analysis. Thus, larger, controlled, multicenter analyses are required to further validate our results.

## 5 Conclusion

Patients with early glottic cancer with close/positive margins after TLM showed equivalent DSS rates and LPRs to those with negative margins; albeit with a lower RFS. Considering that only some of the patients (approximately 20%) developed recurrence and the median time to recurrence for the close/positive margin group exceeded 1.5 years, revision surgery in patients with close/positive margins might be considered overtreatment. Thus, we believe that a follow‐up approach, which avoids unnecessary tissue loss and preserves vocal function, is enough to manage this condition.

## Conflicts of Interest

The authors declare no conflicts of interest.

## Data Availability

The data that support the findings of this study are available on request from the corresponding author. The data are not publicly available due to privacy or ethical restrictions.
